# Neuronatin Promotes Neural Lineage in ESCs via Ca^2+^ Signaling

**DOI:** 10.1002/stem.530

**Published:** 2010-09-24

**Authors:** Hsuan-Hwai Lin, Esther Bell, Dafe Uwanogho, Leo W Perfect, Harun Noristani, Thomas J D Bates, Vladimir Snetkov, Jack Price, Yuh-Man Sun

**Affiliations:** a Institute of Psychiatry, King's College London, Centre for the Cellular Basis of Behaviour LondonUnited Kingdom; b Department of Internal Medicine, Tri-Service General Hospital, National Defense Medical CenterTaipei, Taiwan, Republic of China; c MRC Centre for Developmental Neurobiology, Kings College London, Guy's CampusLondon, United Kingdom; d Department of Asthma, Allergy and Respiratory Science, Franklin-Wilkins Building, King's College LondonLondon, United Kingdom

**Keywords:** Neural development, Embryonic stem cells, Calcium signaling, FGF/Erk pathway, Nnat, BMP pathway

## Abstract

Neural induction is the first step in the formation of the vertebrate central nervous system. The emerging consensus of the mechanisms underling neural induction is the combined influences from inhibiting bone morphogenetic protein (BMP) signaling and activating fibroblast growth factor (FGF)/Erk signaling, which act extrinsically via either autocrine or paracrine fashions. However, do intrinsic forces (cues) exist and do they play decisive roles in neural induction? These questions remain to be answered. Here, we have identified a novel neural initiator, neuronatin (Nnat), which acts as an intrinsic factor to promote neural fate in mammals and *Xenopus*. ESCs lacking this intrinsic factor fail to undergo neural induction despite the inhibition of the BMP pathway. We show that Nnat initiates neural induction in ESCs through increasing intracellular Ca^2+^ ([Ca^2+^]_i_) by antagonizing Ca^2+^-ATPase isoform 2 (sarco/endoplasmic reticulum Ca^2+^-ATPase isoform 2) in the endoplasmic reticulum, which in turn increases the phosphorylation of Erk1/2 and inhibits the BMP4 pathway and leads to neural induction in conjunction with FGF/Erk pathway. STEM CELLS 2010;28:1950–1960

## INTRODUCTION

During mouse embryogenesis, the inner cell mass of the blastocyst differentiates into pluripotent primitive ectoderm and gives rise to a structure known as the epiblast [1]. The epiblast responds to extrinsic signals and generates three primary germ layers: ectoderm, mesoderm, and endoderm [2]. At the stage of neural induction, the ectoderm gives rise to the neuroectoderm (called the neural plate, composed of neuroepithelial cells/neural stem cells [NSCs]) [3]. Knowledge of the mechanism underlying neural induction is primarily derived from studies in *Xenopus*, in which bone morphogenetic proteins (BMPs) act as a decisive inhibitory force during neural induction. This generates the neural default model, which postulates that in the absence of BMP signaling, ectodermal cells will adopt a neural fate [4]. However, the default model remains a debated issue [5,6]. To date, several signaling pathways, including BMP [7–10], FGF [11–17], Ca^2+^ [18,19], Wnt [20–23], and Shh [24] have been shown to play critical roles in neural induction in various species. BMP4, FGFs, Wnt, and Shh exert their regulatory roles in neural induction by binding to their receptors in the cell membrane of ectodermal cells, which in turn trigger intracellular signaling cascades. These factors exert extrinsic influences on ectodermal cells to inhibit or induce neural induction. So far, no intrinsic factor (other than Smad7, if it can be considered as one) [25] has been identified and its role in neural induction is basically unknown.

In a wider study to screen for genes involved in neural induction, we have identified a novel intrinsic neural initiator, Neuronatin (Nnat). *Nnat* is a maternal imprinted gene located on mouse chromosome 2 [26], which encodes a membrane protein in the endoplasmic reticulum (ER) [26] and is predominantly expressed in the developing brain [26,27]. Transcription processing of *Nnat* gene leads to the generation of two alternatively spliced isoforms (α and β mRNA) [28] and their expression patterns suggest that *Nnat* plays a role in brain development [29,30]. However, its precise functions during central nervous system development remain to be elucidated. In this study, we address the role and mechanistic action of Nnat in neural induction and neuronal differentiation using an in vitro mammalian embryonic stem cell (ESC)-derived neural lineage model [31,32], which recapitulates neural development in vivo, and an in vivo *Xenopus* neural induction system. Our findings reveal that Nnat is an intrinsic neural initiator that functions by triggering Ca^2+^ signaling that leads to ESC differentiation toward the neural lineage and also alters neural patterning in *Xenopus*.

## MATERIALS AND METHODS

### Generation of Nnat ES Mutants

46C ESCs (a gift from Prof. A. Smith, Cambridge) were used to create Nnat-knockdown (Nnat-KD) and Nnat-overexpression (Nnat-OE) ESCs. To generate Nnat-KD ESCs, the ESCs were stably transfected with two designed Nnat short hairpin RNA (Nnat-shRNA) pENTR/H1/TO vectors, which were designed to knockdown both *Nnat*α and β isoforms. The sequences of the two oligos were 5′-gcatttactgggtaggattcg-3′ and 5′-gcacacatattcctgccttgc-3′. Three clones were selected for further analysis after 14-day Zeocin (50 μg/ml) selection. As three clones exhibited a similar knockdown level of *Nnat* and have shown a similar phenotype, we designated one clone as Nnat-KD ESCs. To generate Nnat-OE ESCs, the cells were stably transfected separately with the pNEBRX1-Hygro vector (BioLabs, Cheltenham, UK, http://www.neb.com/nebecomm/default.asp) containing a full-length cDNA of *Nnat*α or *Nnat*β, and the cells were under Hygromycin selection (200 μg/ml). The ESC line stably transfected with Nnatα was designated as Nnatα-OE, whereas transfected with Nnatβ as Nnatβ-OE ESCs. The control ESCs were stably transfected with empty pENTR/H1/TO and pNEBRX1-Hygro vectors.

### ESC Monolayer Differentiation

ESCs were differentiated toward the neural lineage using monolayer culture in N2B27 medium as previously described [31]. In general, ESC-derived neural differentiation spans 14 days, which consisted of four stages: ESC (day 0), NSCs (day 6), radial glia-like progenitors (day 10), and neurons (day 14). Cells were collected from those days for immunocytochemical and fluorescence-activated cell sorting (FACS) analysis. As Sox1-eGFP^+^/Nes^+^ NSCs usually appear 2 days after the addition of N2B27 to cells. In the thapsigargin (Tg)- and 2,5-di-*t*-butyl-1,4-benzohydroquinone (BHQ)-rescuing experiments, we administrated the drugs at the same time as N2B27 was first given to cells, whereas in FGF4 and FGF5-rescuing experiments, drugs were administrated 1 day after the cells were changed to N2B27 medium. To determine the effects of the BMP4, FGF, and Erk1/2 signaling pathways on Tg or FGFs rescue of neural induction, ESCs were treated with 25 nM Tg or FGF4 (5 ng/ml; 10 ng/ml for FGF5) in conjunction with either BMP4 (10 ng/ml), PD173074 (100 ng/ml), or 5 μM PD184352. Cells were then collected at 6-day differentiation for analyzing Sox1-eGFP^+^/Nes^+^ NSCs. To determine the effects of BMP4 antagonists on neural induction in Nnat-KD ESCs, the cells were treated with Nog (200 ng/ml), Chrd (300 ng/ml), and Fst (300 ng/ml) alone or in combination as N2B27 was applied to the cells. Cells then were collected at 4-day differentiation for analyzing Sox1-eGFP^+^ NSCs.

### Embryonic Body Formation

ESCs were plated out at 1 × 10^7^ cells/10 cm plate in bacterial plates in ES medium without leukemia inhibitory factor (LIF). Embryonic bodies (EBs) were collected 6 days after differentiation for isolation of total RNA.

### FACS Analysis

Fluorescently labeled cells were FACS sorted and counted using a BD FACSAria (BD Bioscience, Oxford, UK, http://www.bd.com/) according to manufacturers' instructions and procedure described in our previous study [33].

### Intracellular Ca^2+^ Imaging and Recording

For intracellular Ca^2+^ level ([Ca^2+^]_i_), recording cells were cultured to confluence on 13-mm-laminin-coated glass coverslips. Coverslips were incubated in the dark for 40 minutes with either 1 μM calcium green-1/AM or 0.5 μM Fura PE-3/AM. Time course of [Ca^2+^]_i_ responses was recorded using microspectrofluorimeter (Cairn Research, Faversham, U.K.). Cell chamber was continuously perfused with HEPES-buffed physiological salt solution, and ratio of Fura PE-3 emission intensities at excitation wavelengths 340 and 380 nm (*R*_340/380_) was taken as a measure of [Ca^2+^]_i_.

### Quantitative Real-Time Polymerase Chain Reaction

Primer design and experimental procedures were described in our previous study [33]. All expression levels were normalized to cyclophillin levels. All data were performed in duplicate and some were repeated three times.

### Statistical Analysis

Statistical significance was determined using a two-tailed Student's *t* test. *p* values of <.05 and <.01 were considered statistically significant. All results are presented as mean ± SD of the mean from experiments that have been repeated three times.

### Immunocytochemistry

ESCs, NSCs, and neurons were fixed in 3% paraformaldehyde for 20 minutes at room temperature (RT). Antibodies included Oct4 (1:500, AB3209, Chemicon, Hampshire, UK, http://www.applegate.co.uk/all-industry/chemicon-europe-1216260.htm), Nes (1:500, MAB353, Chemicon), Pax6 (1:500, Hybridoma bank, Iowa, USA, http://dshb.biology.uiowa.edu/), RC2 (1:500, Hybridoma bank), NeuN (1:500, MAB377, Chemicon), MAP2 (1:400, ab10588-50, Abcam, Cambridge, UK, http://www.abcam.com/), sarco/endoplasmic reticulum Ca^2+^-ATPase isoform 2 (SERCA2; 1:500, sc-8094, Santa Cruz, Heidelberg, Germany, http://www.scbt.com/), and Nnat (sc-30188, Santa Cruz and ab27266, Abcam). All primary antibody staining was carried out at 4°C overnight. Samples were then stained with appropriate fluorescence-conjugated secondary antibodies at RT for 1 hour and examined under an Axiovision fluorescent microscope aided with an APTOM device (Zeiss).

### *Xenopus* Embryo, Animal Cap Assay, and Microinjection

The RNAs of Nnat isoforms were produced by using the mMessage mMachine SP6 kit (Ambion, Warrington, UK, http://www.ambion.com/). For phenotype analysis, embryos were injected in one cell at the 2-cell-stage with 1 ng of either Nnatα or Nnatβ and left to late neurula or early tadpole stages. For cap assay, embryos were injected either in the ventral marginal zone or animal pole at the one- to two-stage with 1 ng RNA. All procedures were according to our previous study [34].

### Western Blot Analysis

The experiments to investigate the effect of Tg-mediated Ca^2+^ signaling on the phosphorylation of Erk were carried out in Nnat-KD ESCs. For dose-response study, 1 μM, 100 nM, and 25 nM thapsigargin were added to Nnat-KD ESCs in ES medium for 5 minutes and cells were harvested. For time course study, thapsigargin were added to Nnat-KD ESCs in ES medium for different time periods (e.g., 5, 10, 20, and 60 minutes) and cells were harvested. For investigating the involvement of FGF pathway in Tg-mediated p-Erk signaling, the ESCs were pretreated with PD173074 for 2 hours to block FGF pathway, then treated with or without Tg and cells were washed and collected. All collected cells were subjected to Western blot analysis hybridized with p-Erk1/2 antibody (Cell Signaling, Hitchin, UK, http://www.cellsignal.com/). For establishing the effect of Tg on BMP4-mediated p-Smad1, ESCs were pretreated with BMP4 for 1 hour and then treated with Tg for 10, 30, and 60 minutes. Cells were washed and harvested for Western blot analysis hybridized with p-Smad1 antibody (Cell Signaling). Nnat antibody for Western blot analysis was purchased from Abcam (ab27266).

### Co-immunoprecipitation of SERCA and Nnat

Co-immunoprecipitation procedures were based on the Universal magnetic co-IP kit (Active Motif, Rixensart, Belgium, http://www.activemotif.com/). Nnat (sc-30188, Santa Cruz) and SERCA2 (1:500, sc-8094, Santa Cruz) antibodies were used in co-immunoprecipitation (co-IP) assay.

### Chemicals

BMP4, FGF4, follistatin, chordin, and noggin were from R&D System, PD184352 from Axon Medchem (Groningen, Netherlands, http://www.axonmedchem.com/news.html), BHQ and 1,2-Bis (2-aminophenoxy) ethane-N,N,N′,N′-tetraacetic acid tetrakis (acetoxy methyl ester) (BAPTA-AM) from Calbiochem (Abington, UK, http://www.emdchemicals.com/life-science-research/calbiochem/c_PmSb.s1ON3EAAAEj0uBXhFCU), and Calcium Green-1/AM from Invitrogen. The rest of drugs were from Sigma.

## RESULTS

### The Expression Pattern of Nnat During Neural Induction/Differentiation and in Nnat ESC Mutants

**Figure 1:**
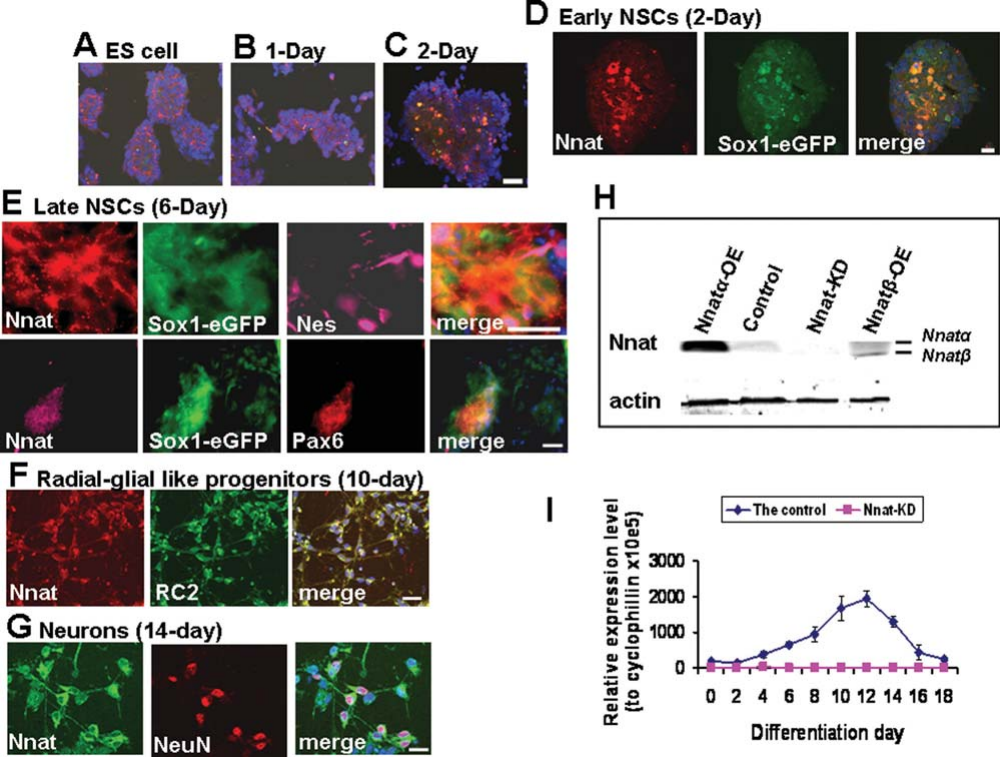
The expression of Nnat during ESC-derived neural differentiation and in the generated Nnat ES mutants. **(A–G):** Immunocytochemical analysis of Nnat expression. Nnat is expressed in a subpopulation of ESCs (before differentiation) **(A)** and 1 day after differentiated is initiated **(B)**. After 2-day differentiation, Nnat is abundantly expressed in Sox1-eGFP^+^ early NSCs **(C)** and at higher magnification **(D)**. As differentiation proceeded, Nnat-positive cells are also costained with the late NSC markers, Nes, and Pax6 **(E)**, radial-glial-like progenitor marker, RC2 **(F)**, and neuronal marker NeuN **(G)**. **(H):** The levels of Nnatα and β in 12-day differentiated cells derived from control and Nnat ESC mutants were measured using Western blot analysis. Nnat ESC mutants are Nnatα-OE, Nnatβ-OE, and Nnat-KD ESCs. **(I):** Confirmation of Nnat-KD throughout 18 days of differentiation by qRT-PCR. Data shown are the mean ± SD (*n* = 2). Scale bar = 50 μm, 20 μm (**[D]**; **[E]**, top panel; and **[G])**. All nuclei were stained with DAPI (blue). Abbreviations: DAPI: 4′,6′-diamidino-2-phenylindole; eGFP, enhanced green fluorescent protein; Nnat, neuronatin; Nnat-KD, Nnat-knockdown; Nnatα-OE, neuronatin αoverexpression; Nnatβ-OE, neuronatin βoverexpression; NSC, neural stem cell; qRT-PCR, quantitative real time-polymerase chain reaction.

In this study, we used Sox1-eGFP knock-in 46C ESCs, in which enhanced green fluorescent protein (eGFP) expression is driven by the *Sox1* promoter and so is concomitantly activated with the expression of an early neuroectodermal marker Sox1. This allows for the appearance of Sox1-eGFP^+^ cells to be used as readout for neural induction [35]. We have found that Nnat was initially expressed in a subpopulation of ESCs and its expression retained in 1-day differentiated cells (Fig. 1A, 1B). As differentiation proceeded, Nnat became abundantly expressed in Sox1-eGFP^+^ neuroectodermal cells (early NSCs; Fig. 1C, 1D), Sox1-eGFP^+^/Nes^+^ or Sox1-eGFP^+^/Pax6^+^ late NSCs (Fig. 1E), RC2^+^ radial-glial like progenitor cells (Fig. 1F), and then neurons (Fig. 1G). However, Nnat expression in progenitor cells and neurons is much weaker than in NSCs. Our data showed that Nnat expression is closely associated with the neural lineage, suggesting that Nnat is involved in aspects of neural development.

*Nnat* consists of two spliced isoforms, a full-length *Nnat*α and a short form *Nnat*β [28]. To address the role of Nnat in neural development, we generated Nnat-overexpressing (Nnatα-OE and Nnatβ-OE) ESCs and Nnat-KD ESCs (knocking down both α and β isoforms). The Nnat ESC mutants were confirmed by the expression levels of Nnatα and β using Western blot analysis (Fig. 1H). Nnatα was detected in control, Nnatα-OE, and Nnatβ-OE ESCs but not in Nnat-KD ESCs. Nnatβ was only detected in Nnatβ-OE ESCs, suggesting that Nnatβ isoform is expressed at a lower level than that of Nnatα isoform. *Nnat* knockdown throughout 18 days of neural differentiation was confirmed by quantitative real-time polymerase chain reaction (PCR; Fig. 1I).

### The Phenotypic Effects of Nnat Dysregulation on ESC-Derived Neural Lineage

**Figure 2:**
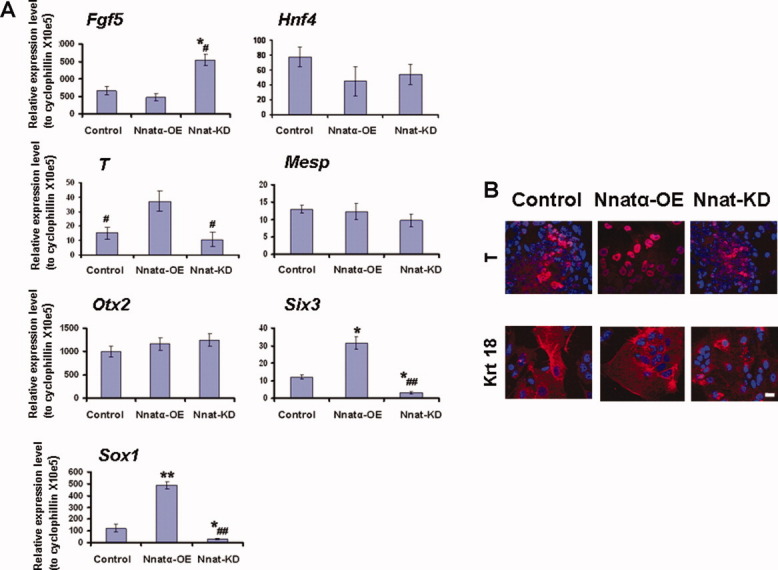
The competence of Nnat overexpressing and knockdown ESCs to give rise to the three primary germ cells. **(A):** Quantitative RT-PCR analysis for markers of the three primary germ cells using RNAs derived from control, Nnatα-OE, and Nnat-KD ESCs using an embryoid body (EB) formation assay. The total RNA from each ESC line was collected from 6-day differentiated EBs and the cDNAs were used to analyze the various cell markers: *Fgf5* (primitive ectodermal cells), *Hnf4* (endodermal cells), *T* and *Mesp* (mesodermal cells), *Otx2* (ectodermal cells), and *Six3* and *Sox1* (neuroectodermal cells). Data shown are the mean ± SD (*n* = 3). *, *p* < .05 and **, *p* < .01, significantly different from the control group; ^#^, *p* < .05 and ^##^, *p* < .01, significantly different from the Nnatα-OE group, two-tailed Student's *t* test. **(B):** ICC analysis of the mesodermal and epidermal cells derived from control, Nnatα-OE, and Nnat-KD ESCs identified by staining with T and Krt18, respectively. Scale bar = 50 μm. All nuclei were stained with DAPI (blue). Abbreviations: DAPI: 4′,6′-diamidino-2-phenylindole; ICC, immunocytochemical; Nnatα-OE, neuronatin α-overexpression; Nnat-KD, neuronatin-knockdown; RT-PCR: real time-polymerase chain reaction.

We first determined whether Nnat affects ESC differentiation into the three primary germ cells using an embryoid body formation assay. Using quantitative PCR analysis for markers of the three germ layers, we found that like control ESCs, Nnatα-OE, and Nnat-KD ESCs can be differentiated toward primitive ectoderm (*Fgf5*) and then to mesoderm (*T* and *Mesp*), endoderm (*Hnf4*), and ectoderm (*Otx2*) fates (Fig. 2A). Nnat-KD ESCs generated significantly higher *Fgf5*-expressing cells, whereas Nnatα-OE ESCs produced more *T*-expressing mesodermal cells. Using immunocytochemical (ICC) analysis, both Nnat ES mutants were shown to produce T^+^ mesodermal cells and Krt18^+^ epidermal cells (Fig. 2B), further suggesting that Nnat dysregulation did not prevent ESCs from differentiating into three primary germ cells. However, the ectodermal cells derived from Nnat-KD ESCs failed to differentiate into neuroectodermal cells (*Sox1* and *Six3*), whereas Nnatα-OE ESCs produced significantly more neuroectodermal cells compared with the control, suggesting that Nnat promotes neural lineage in ESCs.

**Figure 3:**
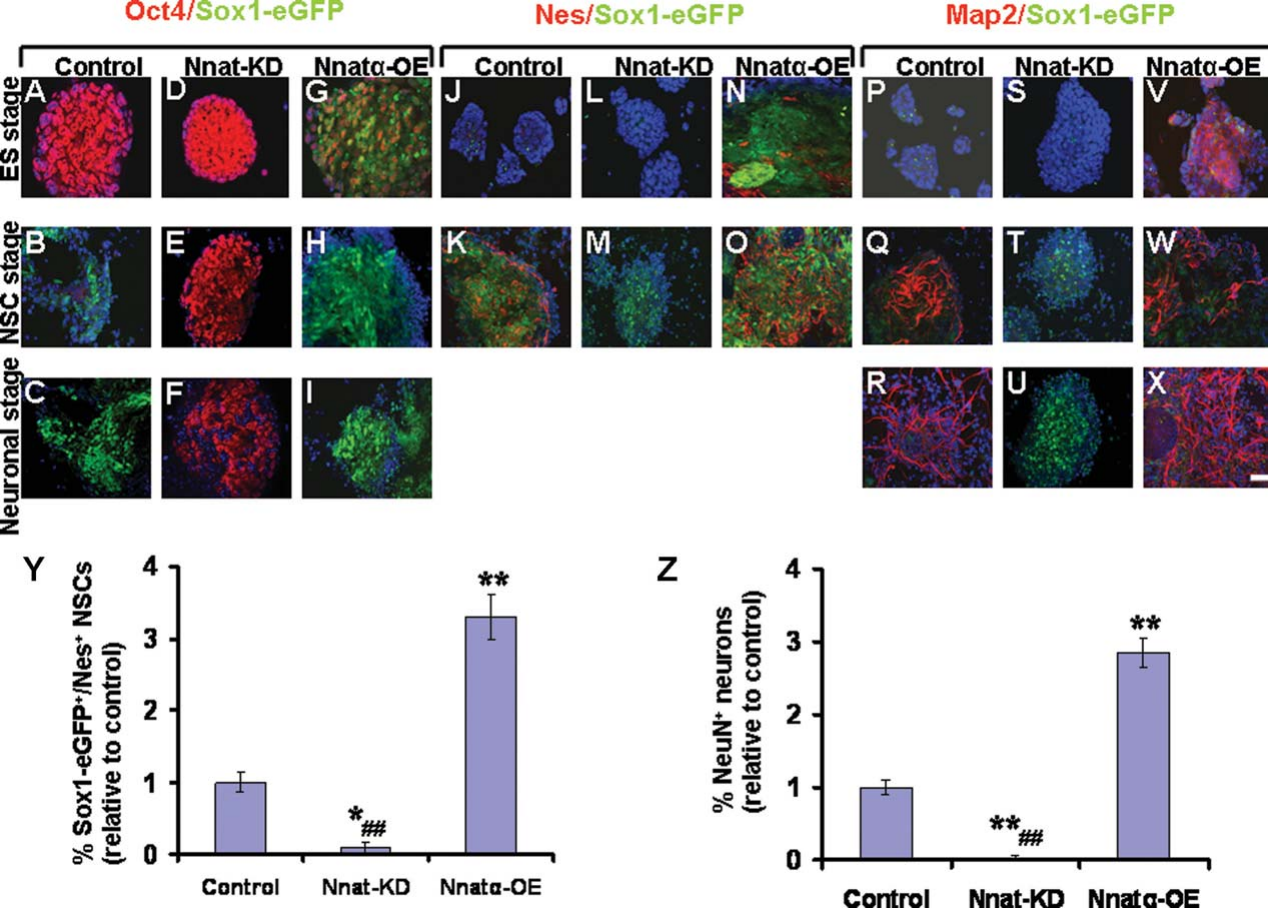
Nnat promotes the neural lineage. The role of Nnat in neural development was examined using an embryonic stem cell (ESC)-derived neural differentiation system over a 14-day time period. **(A–X):** Control, Nnat-KD, and Nnatα-OE ESCs were driven along neural differentiation using monolayer culture in N2B27 medium. Samples were collected for immunocytochemical analysis at ESC stage (before differentiation), NSC stage (6-day differentiation), and neuronal stage (14-day differentiation). The samples were stained with cell-specific markers, Oct4 (red), Nes (red), and Map2 (red), which represent ESCs, NSCs, and neurons, respectively. Scale bar = 50 μm. All nuclei were stained with DAPI (blue). **(Y):** Quantification of Sox1-eGFP^+^/Nes^+^ neural stem cells derived from control, Nnat-KD, and Nnatα-OE ESC using fluorescence-activated cell sorting (FACS) analysis. **(Z):** Quantification of NeuN^+^ neurons derived from control, Nnat-KD, and Nnatα-OE ESC using FACS analysis. Data shown are the mean ± SD (*n* = 3). *, *p* < .05 and **, *p* < .01, significantly different from the control group; ^#^, *p* < .05 and ^##^, *p* < .01, significantly different from the Nnatα-OE group, two-tailed Student's *t* test. Abbreviations: DAPI: 4′,6′-diamidino-2-phenylindole; eGFP, enhanced green fluorescent protein; ES, embryonic stem; Nnat-KD, neuronatin-knockdown; Nnatα-OE, neuronatin α-overexpression; NSC, neural stem cell.

To further corroborate these results, we delineated the phenotypic effects of Nnat dysregulation in neural specification using a monolayer culture. We found that Nnat-KD ESCs exhibited protracted Oct4 expression even after 14 days under neural differentiation conditions (Fig. 3D–3F), whereas the expression of Oct4 in the control cells was significantly reduced by 6-day neural differentiation (at NSC stage; Fig. 3A–3C). In contrast, Nnatα-OE ESCs exhibited less Oct4 staining, concomitant with the precocious expression of Sox1-eGFP (Fig. 3G–3I). As differentiation proceeded, Nnat-KD ESCs generated only a few small rounded Sox1-eGFP^+^ cells (Fig. 3M), which never become Nes^+^. These cells were distinct from those derived from control ESCs (Fig. 3K), which were flatter, larger in size, and Nes^+^ (Supporting Information Fig. S1). Interestingly, the Sox1-eGFP^+^ cells generated from Nnat-KD ESCs failed to differentiate into microtubule-associated protein 2 (Map2^+^) neurons (Fig. 3T, 3U), whereas control ESCs generated numerous Map2^+^ neurons (Fig. 3Q, 3R). Conversely, even in ESC medium, Nnatα-OE ESCs prematurely produced Sox1-eGFP^+^/Nes^+^ NSCs (Fig. 3N) and Map2 stained neuron-like cells (Fig. 3V). The aforementioned data were confirmed by FACS analysis, which showed that Nnatα-OE ESCs generated three times more Sox1-eGFP^+^/Nes^+^ NSCs (Fig. 3Y) and NeuN^+^ neurons (Fig. 3Z) than control, whereas Nnat-KD ESCs displayed few, if any, NSCs and neurons. We also found Nnatβ-OE ESCs exhibited a similar phenotype to that of Nnatα-OE ESCs (data not shown). We demonstrated that the phenotype of Nnat-KD ESCs is not due to an off-target effect. We showed that the restoration of Nnat expression in Nnat-KD ESCs (called Nnat-KD+Nnat ESCs; Supporting Information Fig. S2A) rescued the phenotypic defects in the production of NSCs and neurons in Nnat-KD ESCs to the phenotypes observed in control ESCs (Supporting Information Fig. S2B–S2D). In summary, our results suggest that Nnat predisposes ESCs to differentiation and then initiates neural induction and promotes downstream neuronal differentiation.

### Nnat Affects the Neural Patterning of *Xenopus laevis*

**Figure 4:**
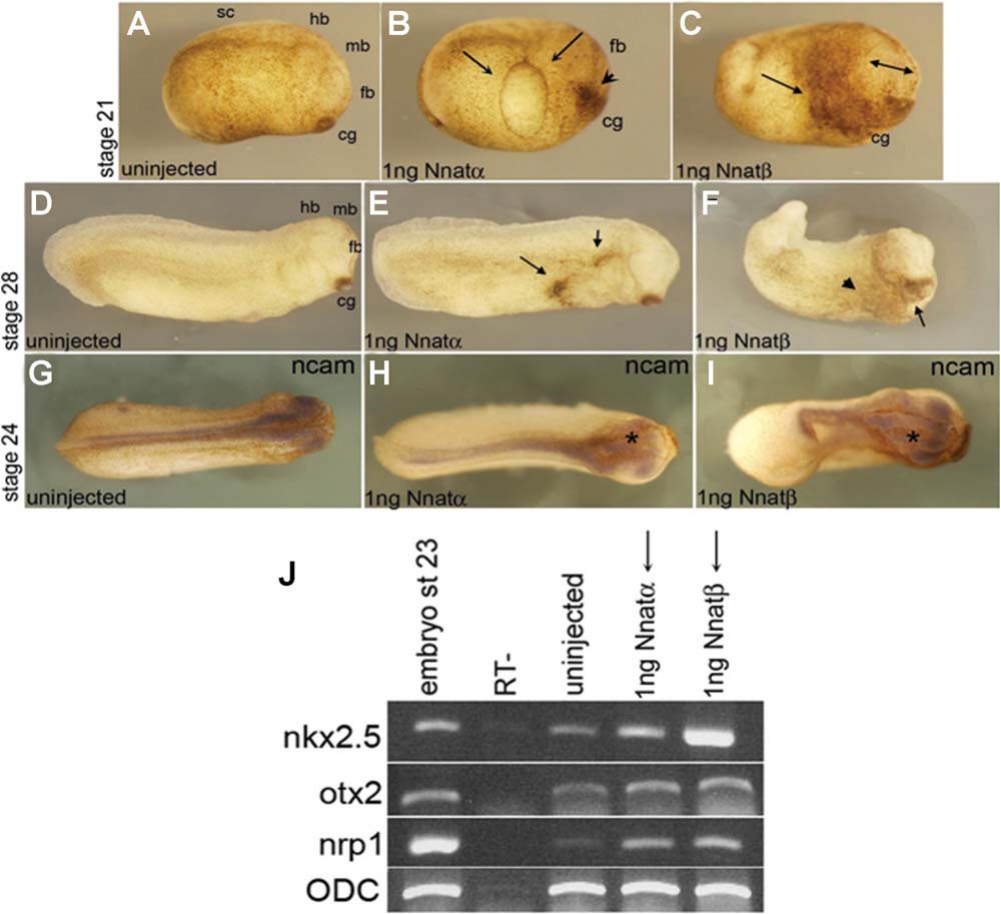
The effects of mouse Nnatα and β on neural patterning in *Xenopus laevis*. **(A–I):** The representative images of *Xenopus* embryos, which were injected in one cell at the two-cell stage with 1 ng of either Nnatα or Nnatβ and the morphology was evaluated at late neurula or early tadpole stages. At neurula stage, **(A)** uninjected control embryo, **(B)** Nnatα injection results in an edema-like structure (see black arrows), and an enlarged cement gland (arrowhead), whereas **(C)** Nnatβ injection not only causes an enlarged cement gland (see arrow) but also an expanded forebrain (double-headed arrow). At early tadpole stages, **(D)** uninjected control embryo, **(E)** Nnatα-injected embryos exhibit an abnormal phenotype with ectopic pigmentations (see arrows) and **(F)** Nnatβ-injected embryos show a distorted and truncated phenotype with an enlarged cement gland (arrow) and a clear expansion of the neural plate. The abnormal neural phenotypes were confirmed by Ncam staining (a pan-neural marker): **(G)** control uninjected embryo, **(H)** Nnatα-injected embryo with noticeable neural plate expansion in the injected side (*), and **(I)** Nnatβ-injected embryo with a profound neural plate expansion in the injected side (*). **(J):** In animal caps assay, injecting either Nnatα or β at one- to two-cell stage leads to increase in the expression of neural markers, nrp1 and otx, and a mesodermal marker nkx2.5, as assayed by RT-PCR. ODC was used as a loading control. Abbreviations: cg, cement gland; fb, forebrain; hb, hindbrain; mb, midbrain; sc, spinal cord; Ncam, neural cell adhesion molecule; Nnatα, neuronatin α; Nnatβ, neuronatin β; ODC, ornithine decarboxylase; RT, no reverse transcriptase; RT-PCR, real time-polymerase chain reaction.

We employed a *Xenopus* neural induction model to examine whether Nnatα and Nnatβ exhibit the same effect in vivo. Our results showed that Nnatα (78% of 104 injections) and Nnatβ (95% of 88 injections) changed the patterning of *Xenopus* embryos, including cement gland formation (Fig. 4A–4F). In particular, Nnatβ injection resulted in distorted and truncated embryos (Fig. 4C, 4F). With regard to neural patterning, Nnatβ exhibited a more potent effect than Nnatα; as seen by the expanded Ncam staining (Fig. 4G–4I). To confirm these results, we also performed animal caps experiments. We found that *nrp1*, a pan-neural marker, and *otx*, a forebrain/midbrain expressed gene, were upregulated in the embryos injected with Nnatα or β (Fig. 4J). Our data suggest that Nnat promotes the neural fate in *Xenopus*. Nnat also increased mesoderm production judging by *nxk2.5* expression (a heart/mesoderm marker; Fig. 4J), which is consistent with our findings in mammalian system that Nnatα-OE ESCs generate significantly more *T*-expressing mesodermal cells (Fig. 2A). Our findings suggest that the role of Nnat in neural induction is conserved from mammals to *Xenopus*.

### Nnat-Mediated Neural Induction Is Via Ca^2+^ Signaling

**Figure 5:**
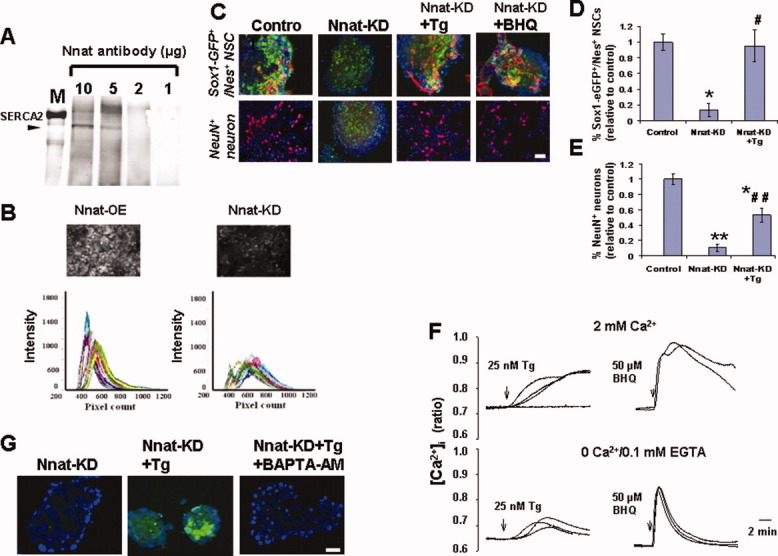
Nnat action is via Ca^2+^ signaling through negatively regulating SERCA2. **(A):** Dose-dependent pull down SERCA2 (100 kDa) by Nnat antibody using co-immunoprecipitation assay, suggesting that Nnat physically interacts with SERCA2. **(B):** Ca^2+^ imaging (top panel) and histograms (bottom panel) generated from Nnat-OE and Nnat-KD ESCs, in which the former exhibits higher [Ca^2+^]_i_ than that of the latter using Ca^2+^ green-1AM dye. **(C):** SERCA blockers, Tg and BHQ, rescue Nnat-KD phenotypes in the production of Sox1-GFP^+^/Nes^+^ (red) NSCs, and NeuN^+^ (red) neurons. Scale bar = 50 μm. **(D):** Quantification of Sox1-eGFP^+^/Nes^+^ NSC derived from control, Nnat-KD ESCs, and Nnat-KD ESCs treated with Tg (Nnat-KD+Tg) using fluorescence-activated cell sorting (FACS) analysis. Data shown are the mean ± SD (*n* = 3). **(E):** Quantification of NeuN^+^ neurons derived from control, Nnat-KD, and Nnat-KD+Tg ESCs using FACS analysis. Data shown are the mean ± SD (*n* = 3). *, *p* < .05 and **, *p* < .01, significantly different from the control group; ^#^, *p* < .05 and ^##^, *p* < .01, significantly different from the Nnat-KD group, two-tailed Student's *t* test. **(F):** Changes in intracellular [Ca^2+^]_i_ levels in Nnat-KD ESCs following Tg or BHQ treatment were measured using the *R*_340/380_ emission intensities ratio of Fura PE-3. **(G):** Inhibition of Tg-rescued neural induction in Nnat-KD ESCs by chelating Tg-increased [Ca^2+^]_i_ using an intracellular calcium chelator BAPTA-AM. Scale bar = 50 μm and all nuclei were stained with DAPI (blue). Abbreviations: BAPTA-AM, 1,2-Bis (2-aminophenoxy) ethane-N,N,N′,N′-tetraacetic acid tetrakis (acetoxy methyl ester); BHQ, 2,5-di-*t*-butyl-1,4-benzohydroquinone; DAPI: 4′,6′-diamidino-2-phenylindole; GFP, green fluorescent protein; eGFP, enhanced green fluorescent protein; Nnat-KD, neuronatin-knockdown; Nnatα-OE, neuronatin α-overexpression; NSC, neural stem cell; SERCA2, sarco/endoplasmic reticulum Ca^2+^-ATPase isoform 2; Tg, thapsigargin.

To elucidate the molecular mechanism of Nnat action, we showed that Nnat physically interacts with SERCA2 using co-IP assay (Fig. 5A) and in situ Proximity Ligation assay using Duolink system (data not shown). SERCA2 is the most dominant SERCA isoform expressed during neural differentiation, suggesting that Nnat exerts its effects by interacting with SERCA2. SERCA is a major component of cellular homeostasis and it maintains physiologically low cytosolic Ca^2+^ levels by pumping [Ca^2+^]_i_ into the ER. To examine whether Nnat acts positively or negatively on SERCA, we monitored [Ca^2+^]_i_ in control, Nnatα-OE, and Nnat-KD ESCs. We found that Nnatα-OE ESCs exhibited uniformly higher [Ca^2+^]_i_ than that of Nnat-KD ESCs (Fig. 5B), whereas the control ESCs showed both high and low levels, corresponding to the “salt and pepper” expression pattern of Nnat in ESCs described earlier (data not shown). Our results show that Nnat increases [Ca^2+^]_i_ by antagonizing SERCA. If this hypothesis is correct, we would expect that the specific SERCA blockers, thapsigargin (Tg) and BHQ, would mimic Nnat actions and be able to rescue the neural defective phenotype of Nnat-KD ESCs. Significantly, we found that Tg and BHQ increased [Ca^2+^]_i_ (Fig. 5F), which in turn rescued the ability of Nnat-KD ESCs to produce NSCs and neurons (Fig. 5C). These data were corroborated by quantitative FACS analysis (Fig. 5D, 5E). Tg rescued the NSC production of Nnat-KD ESCs to the level of control ESCs (Fig. 5D). However, neuronal production was significantly less robust than that of control ESCs (Fig. 5E). This incomplete rescue was possibly due to the fact that only a single dose of Tg was given before neural induction, thus only the immediate functions of endogenous Nnat would be restored. Nnat is also expressed in subsequent stages of neural development (Fig. 1), implying later roles in neurogenesis in addition to the early role in neural induction. We have observed that the increase in the duration or frequency of 25 nM Tg treatment results in cell death.

To further establish that the Tg-mediated rescue of Nnat-KD defects in neural induction was via Ca^2+^ signaling, we assessed whether the blockade of Ca^2+^ signaling, using membrane permeable Ca^2+^ chelator BAPTA-AM, would abolish the rescue by Tg in Nnat-KD ESCs. We found that BAPTA-AM not only prevented the rescue effect of Tg on neural induction (Fig. 5G) but also mitigated neural induction in control and Nnat-OE ESCs (Supporting Information Fig. S3), indicating that Ca^2+^ signaling is a prerequisite for neural specification. Taken together, our results suggest that Nnat acts in a cell autonomous fashion to initiate neural induction via Ca^2+^ signaling by antagonizing SERCA2 in the ER.

### Tg-Mediated Ca^2+^ Signaling Increases the Phosphorylation of Erk1/2 and Cross Talks with FGF/Erk Pathway to Initiate Neural Induction

**Figure 6:**
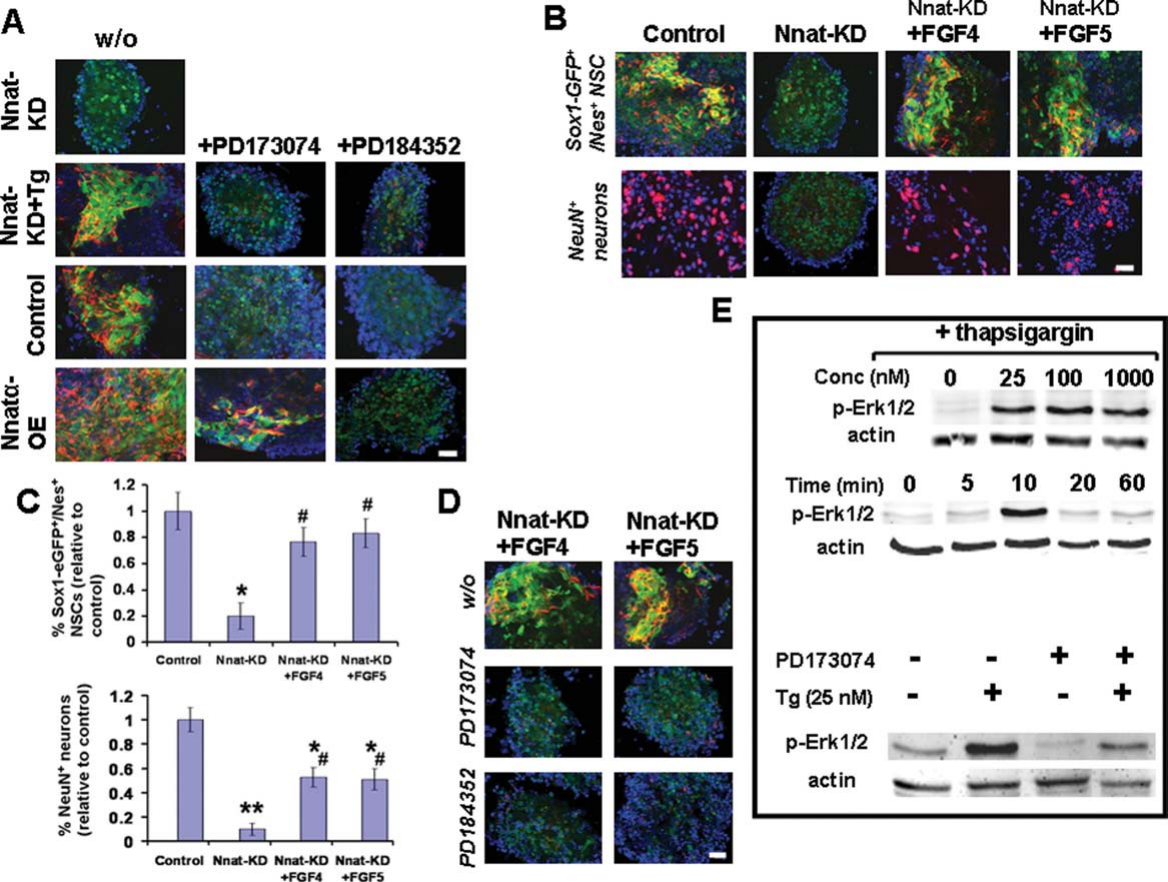
Nnat-mediated Ca^2+^ signaling increases p-Erk1/2 and cross talks with FGF/Erk pathway in neural induction. **(A):** The inhibitory effect of PD173074 (a FGF-R blocker) and PD184352 (a p-Erk1/2 blocker) on neural induction. Without the presence of blockers, Nnat-KD ESCs failed to initiate neural induction (fail to generate Sox1-eGFP^+^/Nes^+^ [red] NSCs), which was rescued by Tg treatment (Nnat-KD+Tg). In the presence of the blockers (+PD173074 or +PD184352), neural induction in control and Nnat-KD+Tg ESCs was abolished, whereas PD173074 partially and PD184352 completely inhibited neural induction in Nnatα-OE ESCs. **(B):** The rescue effects of FGF4 or FGF5 on Nnat-KD phenotype in the production of Sox1-eGFP^+^/Nes^+^ (red) NSCs and NeuN^+^ (red) neurons. **(C):** Quantification of NSC and neuron population generated from control, Nnat-KD, and Nnat-KD ESCs treated with FGF4 (Nnat-KD+FGF4) or FGF5 (Nnat-KD+FGF5) using fluorescence-activated cell sorting analysis. Data shown are the mean ± SD (*n* = 3). *, *p* < .05 and **, *p* < .01, significantly different from the control group; ^#^, *p* < .05, significantly different from the Nnat-KD group, two-tailed Student's *t* test. **(D):** The FGF4 and FGF5 rescue of neural induction in Nnat-KD ESCs were abrogated by the presence of PD173074 or PD184352. **(E):** Increase in the phosphorylation of Erk1/2 after 5 minutes treated with various concentrations of Tg (top panel), the time points of the increment with 25 nM Tg treatment (middle panel), and the effect of PD173074 on Tg-induced p-Erk1/2 (bottom panel). Scale bar = 50 μm and all nuclei were stained with DAPI (blue). Abbreviations: DAPI, 4′,6′-diamidino-2-phenylindole; eGFP, enhanced green fluorescent protein; FGF4, fibroblast growth factor 4; FGF5, Fibroblast growth factor 5; GFP, green fluorescent protein; Nnatα-OE, neuronatin α-overexpression; Nnat-KD, neuronatin-knockdown; NSC, neural stem cell; Tg, thapsigargin; w/o, without.

According to the recent emerging consensus on the regulation of neural induction, the FGF/Erk pathway is one of main regulatory routes in vertebrates [16,17,36–38]. We wished to determine the functional relationship between Ca^2+^ signaling and FGF/Erk pathway in Nnat-mediated neural induction. First, we examined whether the FGF/Erk pathway is involved in Tg rescue of neural induction in Nnat-KD ESCs using a biochemical approach. We found that the Tg rescue was abrogated by both PD173074 and PD184352 (Fig. 6A), which are specific blockers for FGF-R and p-Erk1/2, respectively. These blockers also abolished neural induction in control ESCs, whereas PD173074 partially and PD184352 completely blocked neural induction in Nnatα-OE ESCs (Fig. 6A). This suggests that both the FGF and p-Erk pathways are involved in Nnat-directed neural induction but that the p-Erk signaling is the most crucial pathway. We then assessed whether exogenous FGFs could rescue the neural defective phenotypes in Nnat-KD ESCs. We found that treating Nnat-KD ESCs with either FGF4 or FGF5 restored the ability of these cells to produce NSC and neurons to the levels seen with Tg-treatment (Fig. 6B, 6C) and these effects were also eliminated by PD173074 and PD184352 treatment (Fig. 6D). Intriguingly, we also discovered that the Tg-mediated Ca^2+^ signaling cross talks with the FGF/Erk pathway at the p-Erk level. Treating Nnat-KD ESCs with 25 nM Tg, the dose at which Tg rescued the neural phenotypes of Nnat-KD ESCs (Fig. 5C), directly increased the phosphorylation of Erk1/2 within 10 minutes (Fig. 6E) without affecting the mRNA levels of FGF4, FGF5, Raf, Erk1, and Erk2. This Tg-mediated increase in p-Erk1/2 was not abolished by PD173074 treatment (Fig. 6E), indicating that the increase in p-Erk1/2 was independent of FGF signaling. Collectively, our results suggest that during neural induction the FGF/Erk pathway co-operate, either by acting downstream from or in parallel to, Nnat-mediated Ca^2+^ signaling. The Erk1/2 signaling may be a key player in neural induction by acting as a convergent point between FGF- and Nnat-mediated signaling.

### Nnat-Mediated Ca^2+^ Signaling Suppresses the Expression of BMP4 and Its Direct Target Genes

**Figure 7:**
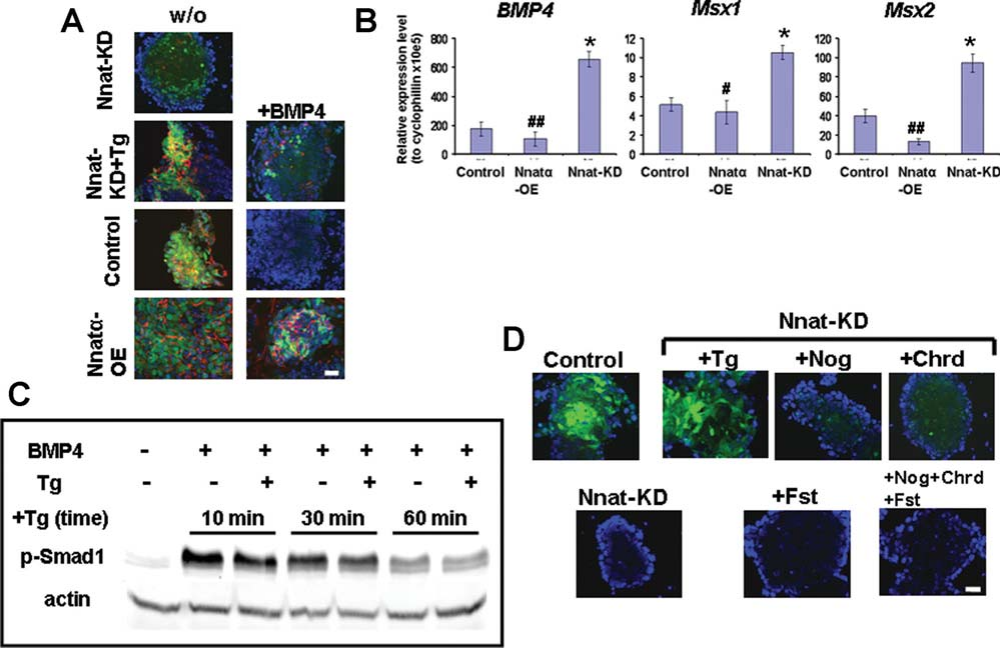
Nnat-mediated Ca^2+^ signaling interacts with BMP4 pathway in neural induction by suppressing the transcription of BMP4 and its target genes. **(A):** The inhibitory effect of BMP4 on neural induction. Without the presence of BMP4, Nnat-KD ESCs failed to generate Sox1-eGFP^+^/Nes^+^ (red) neural stem cells, which was rescued by Tg treatment (Nnat-KD+Tg). In the presence of BMP4 (+BMP4), neural induction in control and Nnat-KD+Tg ESCs was abolished, whereas BMP4 only partially inhibited neural induction in Nnatα-OE ESCs. **(B):** Gene expression profiles of *BMP4* and its target genes, *Msx1* and *Msx2* in ESCs show that the expression of those genes is suppressed in Nnat-mediated Ca^2+^ signaling. Data shown are the mean ± SD (*n* = 3). *, *p* < .05, significantly different from the control group; ^#^, *p* < .05 and ^##^, *p* < .01, significantly different from the Nnat-KD group. **(C):** The effect of Tg on BMP4-mediated C-terminal phosphorylation of Smad1 at indicated duration of Tg treatment. **(D):** Inhibition of BMP pathway by antagonists, Nog, Chrd and Fst, does not induce neural induction in Nnat-KD ESCs. Control and Nnat-KD ESCs were driven along neural differentiation. At 4-day differentiation, control ESCs generate many Sox1-eGFP^+^ neuroectodermal cells, whereas Nnat-KD ESCs fail to produce neuroectodermal cells. The failure of neural induction in Nnat-KD ESCs is rescued by Tg treatment, but not by BMP antagonists. Scale bar = 50 μm and all nuclei were stained with DAPI (blue). Abbreviations: DAPI, 4′,6′-diamidino-2-phenylindole; BMP4, bone morphogenetic protein 4; Nnatα-OE, neuronatin α-overexpression; Nnat-KD, neuronatin-knockdown; Tg, thapsigargin; w/o, without.

We further investigated whether Nnat-mediated signaling also cross talks with the BMP pathway, which is considered to be the principal signaling pathway in the default model of neural induction [6]. We found that the Tg rescue of neural induction in Nnat-KD ESCs was inhibited by BMP4 (Fig. 7A). However, while BMP4 abolished neural induction in control ESCs, BMP4 only partially inhibited neural induction in Nnatα-OE ESCs (Fig. 7A), suggesting that Nnat can counteract or override the BMP4-directed inhibition of neural induction. We then sought to determine where the cross talk occurs between Nnat-mediated Ca^2+^ signaling and the BMP4 pathway. We found that Nnat-mediated Ca^2+^ signaling significantly suppressed the expression of BMP4 and its direct target genes, *Msx1* and *Msx2* (Fig. 7B). However, we did not find that Tg-mediated Ca^2+^ signaling affected BMP4-mediated phosphorylation of Smad1 at the C-terminus (Fig. 7C). Whether the Tg-mediated Ca^2+^ signaling regulates the phosphorylation of the linker region of Smad1, which has been shown to antagonize the BMP4/Smad1 signaling, remains to be determined. Our results suggest that Nnat-mediated Ca^2+^ signaling inhibits the BMP4 pathway in neural induction at least by suppressing *BMP4* expression. As the BMP pathway is the decisive influence in neural induction in the default model, we wished to test whether, in the absence of the Nnat-directed intrinsic cue, inhibition of BMP is sufficient to induce neural induction in Nnat-KD ESCs. Our findings showed that Nog, Chrd, and Fst, which are antagonists of BMP pathway, either alone or in combination, could not induce neural induction in Nnat-KD ESCs (Fig. 7D), whereas the combination of antagonists prevented the inhibitory effect of BMP4 on neural induction in control ESCs (data not shown). This indicates that the Nnat-led intrinsic cue is crucial in neural induction; without this intrinsic cue, ESCs fail to initiate neural induction despite the inhibition of BMP pathway.

## DISCUSSION

We show that Nnat is initially expressed in a subpopulation of ESCs and then in the neural lineage as differentiation proceeds, which correlates with the expression profile of Nnat in vivo [29–30,39]. We further demonstrate that in loss-of-function studies, Nnat-KD ESCs fail to produce neuroectodermal cells and neurons using both monolayer culture and embryoid body formation approaches, despite the fact that they are differentiated into primary germ cells in embryoid body assay. Conversely, in gain-of-function studies, Nnat-OE ESCs precociously produce neuroectodermal cells and neuron-like cells even in pluripotent medium (containing LIF and serum) and generate three times more neuroectodermal cells and neurons than those derived from control ESCs in differentiation medium. Our findings suggest that Nnat possesses the ability to push ESCs out of the “ground state” and initiate differentiation and then drive the ESCs toward a neural fate. This is supported by our observations that Nnat-OE ESCs express low levels of the pluripotent marker Oct4 and are predisposed to differentiation. In contrast, Nnat-KD ESCs protract the expression of Oct4 even in differentiation media. Moreover, our unpublished data show that Nnat-KD ESCs are refractory to BMP4-induced non-neural differentiation but respond to BMP4-driven differentiation after treatment with Tg, which mimics the action of Nnat. Intriguingly, we find that Tg-mediated Ca^2+^signaling in Nnat-KD ESCs results in an increase in the phosphorylation of Erk1/2. As the Erk1/2 MAP kinase pathway has been shown to promote ESCs differentiation [14,15,40,41], we propose that Ca^2+^-mediated Erk1/2 signaling can, at least in part, contribute to the differentiation-prone phenotype observed in Nnat-OE ESCs. Our findings suggest that Nnat not only triggers ESC differentiation but also sequentially promotes neural fate.

We establish that the Nnat-initiated neural induction results from Nnat-mediated increase in [Ca^2+^]_i_ from internal stores by potentially antagonizing SERCA2 in the ER. Furthermore, we also show that both mouse Nnat isoforms exhibit a similar role in in vivo neural patterning in *Xenopus*. Our results are concomitant with previous findings in frogs, which showed that Ca^2+^ is a potent neural initiator in animal caps and embryos [18,19,42–45]. In *Xenopus*, an increase in [Ca^2+^]_i_ due to an efflux of Ca^2+^ from internal stores triggers neural fate in the dissociation of animal caps [45]. The importance of internal store-mediated Ca^2+^ signaling in early brain development is also shown by Tg-induced Holoprosencephaly in Zebrafish [46]. Our study is the first to show that Ca^2+^ signaling plays a crucial role in neural induction in mammals and also the first to demonstrate that Nnat promotes neural lineage in mammals and *Xenopus*. Interestingly, Nnat also promotes mesodermal cells in the mouse and *Xenopus* models. It has been shown that the Spemann's organizer induces neural tissue from dorsal ectoderm and dorsalizes lateral and ventral mesoderm in *Xenopus*, suggesting that mesoderm plays a role in neural induction [7]. However, we do not think that the Nnat-mediated neural induction in *Xenopus* is through mesodermal interactions. This is supported by our observations that Nnat initiates neural induction in control and Nnat-OE ESCs without the presence of mesodermal cells as judged by the absence of T^+^ cells. Moreover, Nnat-KD ESCs do produce mesodermal cells but fail to generate neuroectodermal cells in an embryoid body formation assay.

We further show that Ca^2+^-mediated Erk1/2 signaling may be responsible for Nnat-initiated neural induction in ESCs. We explore the mechanism of Nnat action in neural induction by employing Tg to mimic the action of Nnat. Our findings reveal that Tg-mediated Ca^2+^ signaling directly increases the phosphorylation of Erk1/2. The p-Erk1/2 signaling pathway has been shown to be the critical pathway in FGF-, RA-, and Syndecan 4-regulated neural induction [13–17,47]. Our findings suggest that Nnat-initiated neural induction may occur in part via p-Erk1/2 signaling. This is substantiated by our findings that the blockage of p-Erk1/2 by mitogen-activated protein kinase/extracellular signal-regulated kinase inhibitor PD184352 eliminates the Tg- and Nnat-initiated neural induction in Nnat-KD and Nnatα-OE ESCs, respectively. Although PD184352-treated ESCs have been reported to be retained in the “pluripotent state” [14], we have observed that around 30% of PD184352-treated cells have differentiated phenotypes (big, flat, and low Oct4 expression) but are not Sox^+^ neuroectodermal cells, suggesting that blocking p-Erk1/2 signaling by PD184352 inhibits neural induction. Taken together, Nnat-mediated Ca^2+^ signaling may increase p-Erk1/2, which in turn leads to neural specification in ESCs. However, we cannot rule out that Nnat-mediated Ca^2+^ signaling may also trigger other pathways to regulate neural induction.

To date, a body of evidence suggests that in addition to BMP inhibition, other signals are required for neural induction, especially the FGF pathway. FGF signaling has been shown to play a pivotal role in the acquisition of neural fate in *Xenopus*, chick, Ciona, and mammals [11,12,14,15,48–50]. Further studies showed that the FGF/Erk pathway is the principal signaling in regulating the neural fate in mouse ESCs [15–17] and *Xenopus* [13]. We show that Nnat-mediated Ca^2+^ signaling interacts with FGF/Erk signaling to regulate neural induction. Our findings reveal that Tg-mediated neural induction in Nnat-KD ESCs and neural induction in control ESCs are abrogated by the FGF-R inhibitor PD173074, whereas this inhibitor only partially blocks Nnat-initiated neural induction in Nnatα-OE ESCs. Moreover, FGF4 or FGF5 treatment rescues the failure of neural induction in Nnat-KD ESCs, despite the cells expressing normal levels of FGF4 and significantly higher levels of FGF5. We also show that Tg-mediated Ca^2+^ signaling directly increases p-Erk1/2 independently of the FGF pathway. Collectively, our findings suggest that the FGF/Erk signaling pathway is involved in neural induction by acting in co-operation with Ca^2+^-mediated p-Erk signaling and the overall levels of p-Erk1/2 produced by these pathways are critical for neural induction. This explains why normal ESCs, which possess intact FGF/Erk and putative Nnat-mediated Erk signaling, are able to initiate neural induction. Whereas, Nnat-KD ESCs containing presumably just FGF/Erk signaling, but without Nnat-mediated Erk signaling, need exogenous FGFs to boost the p-Erk levels to that required for neural induction to occur. This intertwined relationship between Ca^2+^ and FGF signaling in neural induction is also demonstrated in *Xenopus*, in which FGF4 directs Ca^2+^ signaling to control early neural genes by activating the dihydropyridine-sensitive Ca^2+^ channels [50].

Neural induction is controlled by extrinsic influences containing positive and negative cues. We show that BMP4 abolishes Tg-initiated neural induction in Nnat-KD ESCs and neural induction in control ESCs. However, BMP4 only partially inhibits neural induction in Nnatα-OE ESCs, suggesting that Nnat promotes neural lineage in ESCs not only by providing a positive influence on neural induction (discussed earlier) but also counteracting BMP4 inhibition. We show that Nnat-mediated Ca^2+^ signaling antagonizes the BMP4 inhibition of neural induction by repressing BMP4 transcription. Intriguingly, we were unable to restore neural induction in Nnat-KD ESCs, despite antagonizing the BMP pathway by the combined treatment with Nog, Chrd, and Fst. This is not due to the treated cells remaining in a pluripotent state, because we observed that the treated cells show an enlarged and flattened phenotype and express low Oct4, which are indications of cell differentiation. Our findings suggest that there are other inhibitory influences in addition to BMP signaling in neural induction.

## CONCLUSION

As the seminal experiment carried out by Spemann and Mangold in 1924 [51], a generally held view in the regulation of neural induction is that it is primarily governed by extrinsic influences (cues) via either autocrine or paracrine fashions acting on ectodermal cells. What roles, if any, do intrinsic cues have in the neural specification of ectodermal cells? In this study, we demonstrate that a novel intrinsic factor, Nnat, plays a decisive role in neural induction in ESCs and neural patterning in *Xenopus*. We propose that Nnat promotes neural lineage in ESCs by increasing [Ca^2+^]_i_ via antagonizing SERCA2, which in turn increase p-Erk1/2 and suppresses *BMP4* transcription, leading to neural induction in co-operation with the FGFs/Erk pathway. In conclusion, our findings provide a new dimension in understanding the mechanisms underlying neural induction through the aspect of an intrinsic control. We propose that neural induction is controlled by both extrinsic and intrinsic factors. Extrinsic factors consist of negative (e.g., BMP4 and others) and positive (e.g., FGFs and others) influences. Nnat is a novel intrinsic factor that initiates neural induction by promoting positive cues and inhibiting negative cues.

## Disclosure of Potential Conflicts of Interest

The authors indicate no potential conflicts of interest.
