# Supplementary figures and images for: Neuronatin Promotes Neural Lineage in ESCs via Ca2+ Signaling
Source: Stem Cells. 2010 Sep 24;28(11):1950–60. doi: 10.1002/stem.530 (PMC3003906; doi:10.1002/stem.530)

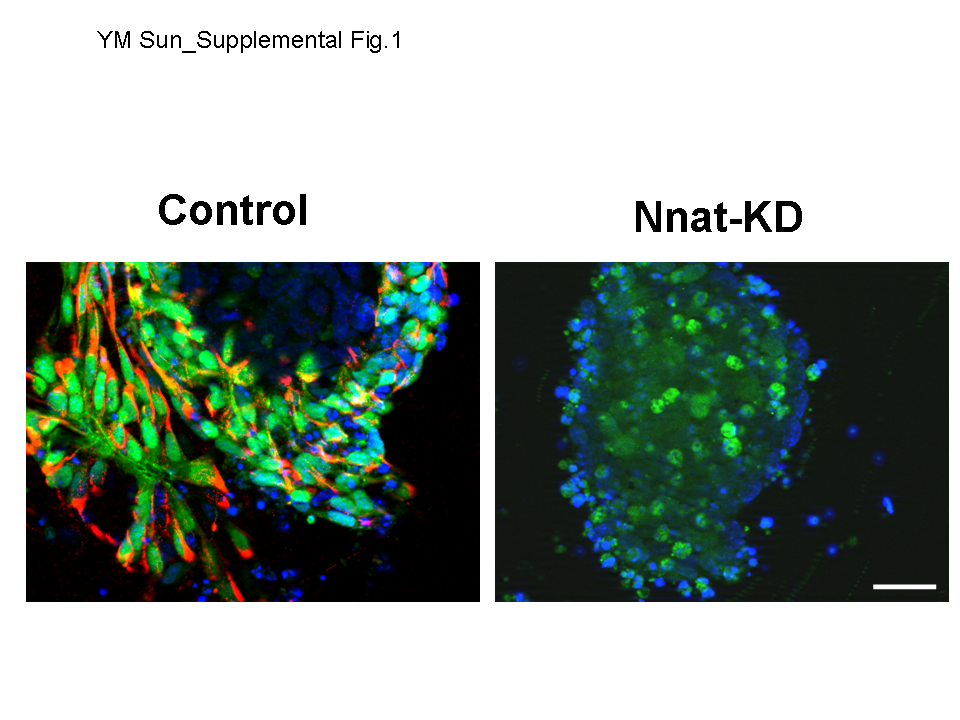

Supplement: Supplementary file 1 [file stem0028-1950-SD1.tif]

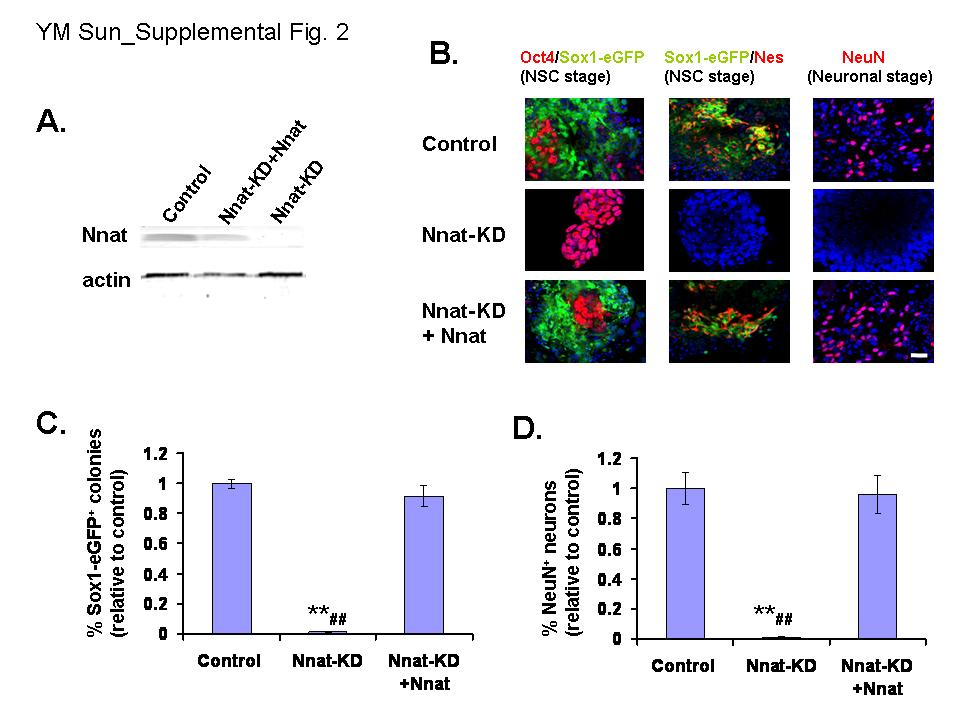

Supplement: Supplementary file 2 [file stem0028-1950-SD2.tif]

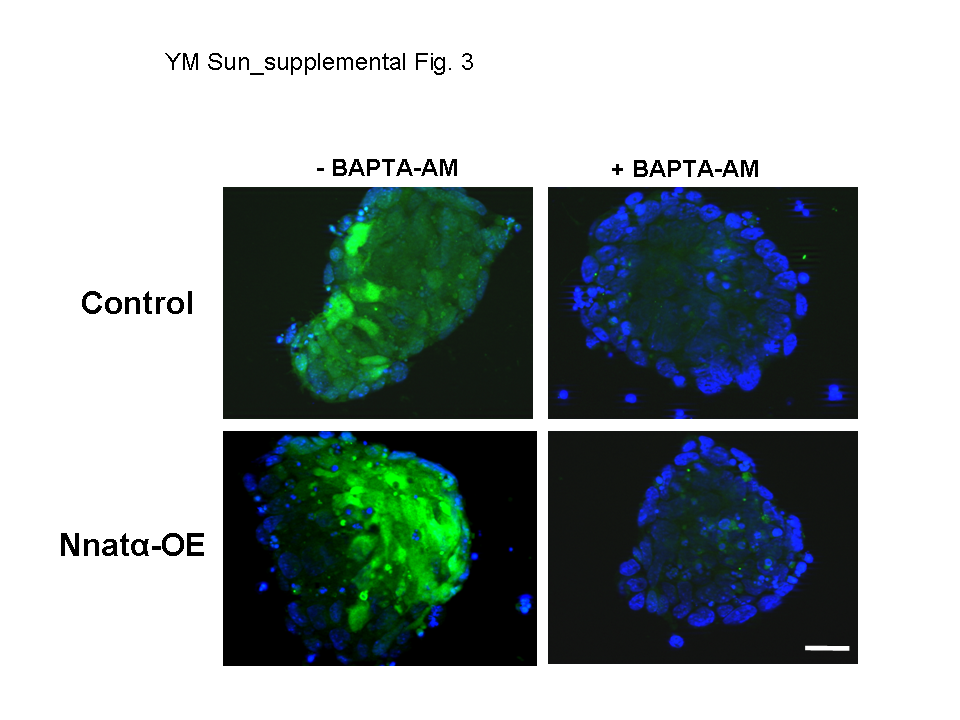

Supplement: Supplementary file 3 [file stem0028-1950-SD3.tif]
